# A bibliometric analysis of publications on burn sepsis using VOSviewer

**DOI:** 10.3389/fmed.2022.971393

**Published:** 2022-09-14

**Authors:** Zhi Cao, Yu Zhang, Jin-Hua Luo, Wen-Qiang Liao, Xing Cheng, Jian-Hua Zhan

**Affiliations:** ^1^Department of Burns, The First Affiliated Hospital of Nanchang University, Nanchang, China; ^2^Medical Innovation Center, The First Affiliated Hospital of Nanchang University, Nanchang, China

**Keywords:** burn sepsis, bibliometric analysis, VOSviewer, direction, Web of Science

## Abstract

**Background:**

Sepsis is one of the most common complications in burn patients and causes high morbidity, especially in those with severe burns. Nevertheless, there are no formal criteria for diagnosing and treating burn sepsis. Therefore, this bibliometric analysis is applied to reveal research trends in this field and predicts its possible hot spots.

**Methods:**

We screened relevant literature on burn sepsis that met the inclusion criteria of the Web of Sciences (WOS) database and analyzed publication trends and research hot spots in related fields using VOSviewer software.

**Results:**

From 1981 to 2022, we screened 2,486 documents that met the requirements and analyzed them bibliometrically. The American scholar Herndon DN had a much higher h-index [47] than other authors. Most published, cited, and h-indexed publications are from the USA (Np: 1193, Nc: 42154, H: 98). The second most publishing country is China, but the second most cited and h-indexed country is Germany. Burns also outperforms other journals in this field (Np: 376, Nc: 8019, H: 46). “Biomarkers” is a newly emerging keyword (cluster “clinical research,” APY was 2018.16), and clinically relevant research in burn sepsis maybe a future research trend.

**Conclusions:**

Sepsis in burn patients has unique pathophysiological characteristics and the general diagnostic criteria for sepsis lack specificity. Consequently, we must establish a database and construct an intelligent predictive model to help achieve a more individualized and precise early diagnosis and treatment of burn sepsis. This may also be an important development direction for future research in this field.

## Introduction

Burns are one of the most common and devastating forms of trauma, and 75% of deaths in patients with severe burns exceeding 40% of the total body surface area (TBSA) are associated with sepsis from burn wound infections and other infectious complications or inhalation injury (1). Initially, we believed that the main cause of death in burn patients who passed through the shock phase was multiple organ dysfunction syndromes (MODS), which directly respond to sepsis after burn injury. Burn patients lose the skin, which is the major barrier against external bacterial infectious attack, resulting in infection vulnerability that can induce sepsis. In addition, this potentially life-threatening infection leads to inadequate tissue perfusion, inflammatory and stress reactions, a prolonged hypermetabolic response, and even sequential multiorgan failure. They are at risk of sepsis and MODS, at least as long as the wound remains open (2). However, the survival rate of patients with post-burn sepsis has not improved significantly over the past decades. Due to these frightening statistics, there have been efforts to improve the salvage rate of post-burn sepsis. Even though more studies have been conducted to investigate this phenomenon, no standardized criteria remain for diagnosing and treating burn sepsis. Therefore, this research aims to comprehensively analyze the current state of sepsis and burn research using the Web of Science (WOS). We applied bibliometric analysis to reveal research trends in this field and provide new directions for burn sepsis.

WOS search platform is an important scientific citation index database, recognized as the most authoritative indexing tool for scientific and technical literature worldwide (3), and the system of SCI citation search is unique, which can evaluate the educational value of articles from the perspective of literature citation and quickly and easily set up a reference network of research topics (4).

Bibliometrics is the cross-cutting science that quantitatively analyzes all knowledge carriers using mathematical and statistical methods. It is a comprehensive knowledge system that integrates mathematics, statistics, and bibliography while emphasizing quantification. Bibliometrics is a convenient method to estimate trends in scientific archives and reveal key research directions by analyzing the characteristics of databases and literature (5, 6). The findings of bibliometric analysis have been applied to various medical fields, including gynecology, orthopedics, ophthalmology, and basic medicine (7–9). However, there remains a lack of bibliometric studies on burn sepsis. Therefore, this study aimed to systematically analyze the research on burn sepsis to identify research trends and hotspots in this field.

## Methods

The search database was WOS database, the search time was March 27, 2022, and the search formula used was as follows: TS = [sepsis OR (septic shock)] AND TS = (burn) AND DOP = (1981-01-01/2022-03-27) AND LA = (English). Articles and review articles written in English only were screened among various publication types. The number of articles that met the criteria was 2,486. The search results were exported as plain text files. The exported information was a complete record, including year of publication, language, journal, title, author, affiliation, keywords, document type, abstract, citation count, etc. The metric package was imported, and VOSviewer analyzed the data. v.1.6.18 (Center for Science and Technology Studies, CWTS, Leiden University, based on JAVA).

### Bibliometric analysis

Bibliometric indicators included the volume of literature, the authors (individual, organization, or country), keywords, the number of citations, and so on. In general, productivity was represented by the number of publications (Np), and the number of citations without self-citations (Nc) was used to measure influence. In addition, citations reflected a general trend. The h-index unified productivity and impact by finding the threshold that connected Np and Nc (10). In other words, A researcher's H-index is defined as having at most H papers that have been cited at least H times each. It also can be extended to describe the impact of publication output of a country, region, institution, or journal (11).

VOSviewer can map and visualize keyword networks related to sepsis and burns. For example, an average year of publication (APY) was used to quantify the relative novelty of a keyword, and link strength can represent the relevance of these items in these networks (12). WOS search platform provides representative citation reports generated by built-in analysis tools, which include Np, Nc, and citations (3).

## Results

### Analysis of thesis on burn sepsis

**Figure 1:**
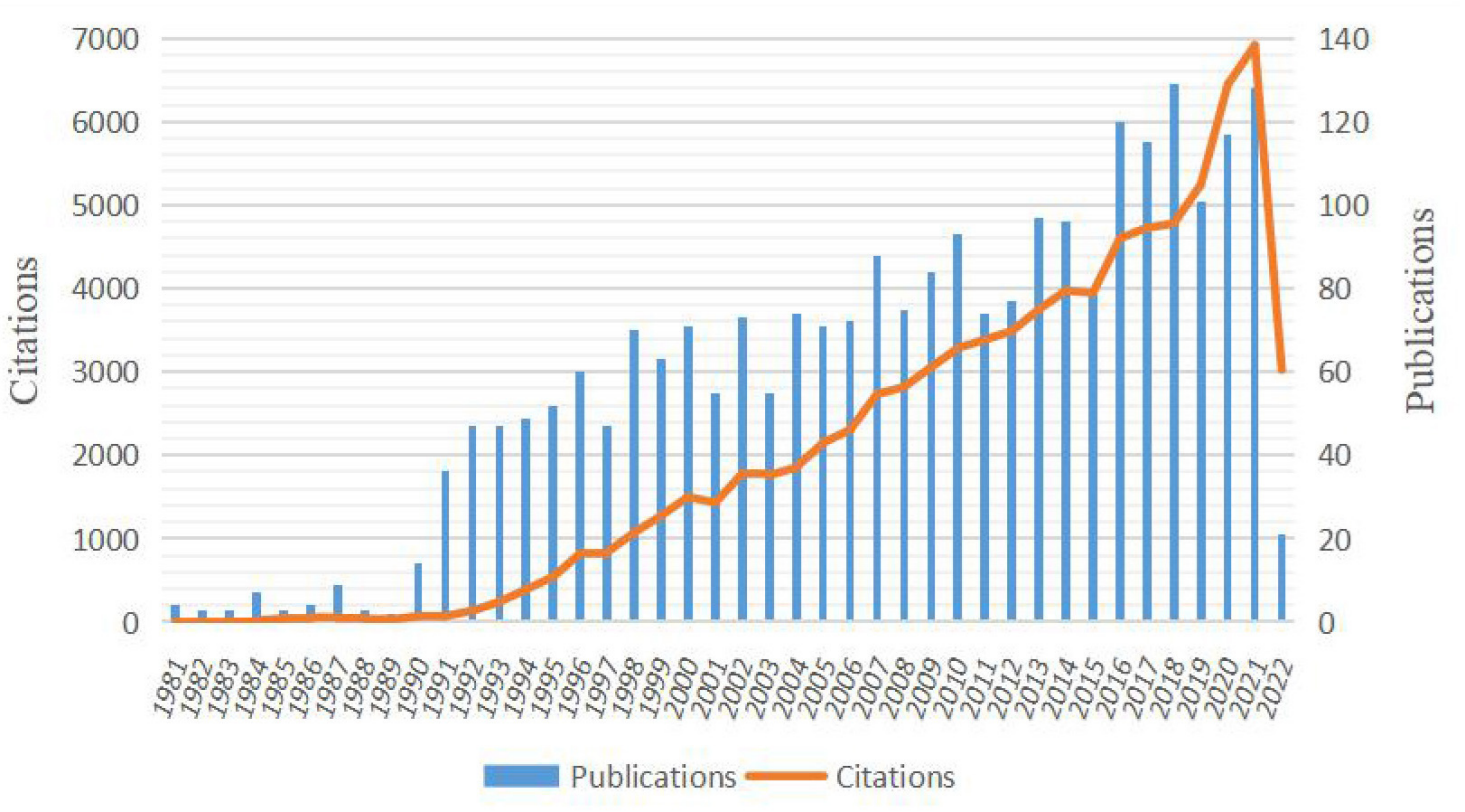
Times cited and publications over time from total documents.

We searched for articles matching the search formula through WOS website. From January 1, 1981 to March 27, 2022, 2,669 English-language articles were searched, including 2,175 (81.5%) research articles and 311 (11.7%) review articles. As depicted in Figure 1, the number of publications grew fastest in the last decade compared to 40 years, accounting for nearly half (46.4%) of the total literature searched. The number of citations in the last decade is also the fastest growing. More interestingly, the citation number was growing faster than the publication number. For the 2,486 documents that met the requirements, citations were 83,119 times, with an average of 33.21 citations per document and an overall h-index of 120.

### Analysis of authors

**Figure 2:**
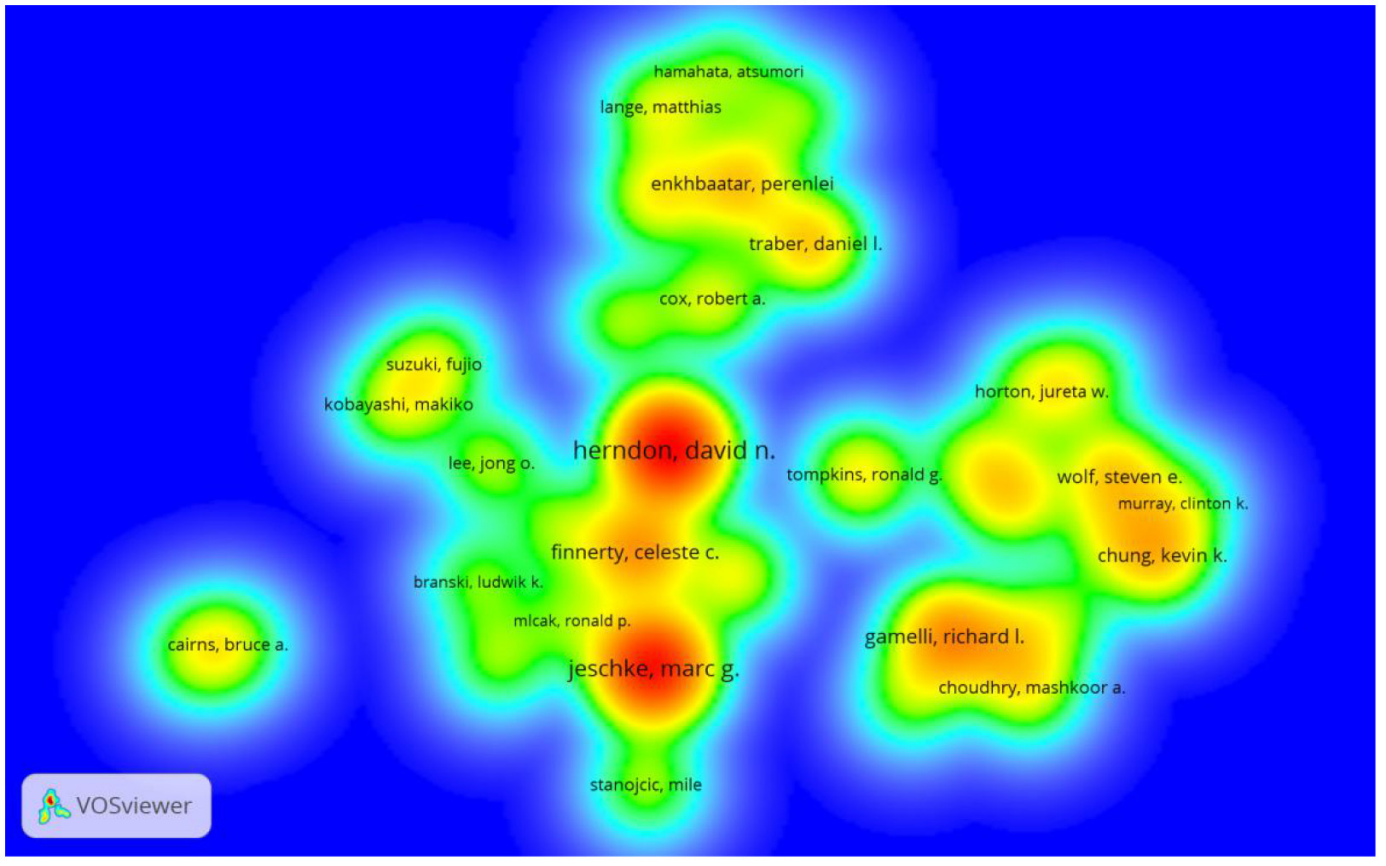
Authors density visualization map according to co-authorship affiliations. Seventy-four authors were included in the map who have at least ten papers. The color represents the number of co-authored documents. Red indicates a higher frequency of occurrence, while green indicates a lower frequency of occurrence.

Table 1 lists the top 10 fruitful authors. They published 535 papers, accounting for 21.52% of all papers. Herndon DN from the US was ranked first in the field of burn sepsis research, followed by Jeschke MG from Canada and Gamelli RL from the US. As revealed in Table 1, Herndon DN had a significantly higher Nc and h-index. In addition, most of the top 10 authors are from the US (7) or China (2). We can also identify from the density visualization map in Figure 2 that Herndon DN and Jeschke MG contributed the most in this field.

**Table 1:** Top 10 active authors.

**Authors**	**Countries**	**Np**	**Nc**	**h-index**
Herndon DN	USA	137	6752	47
Jeschke MG	Canada	87	3521	34
Gamelli RL	USA	61	1994	32
Horton JW	USA	45	1997	24
Wolf SE	USA	37	2083	23
Sheng ZY	China	35	702	16
Traber DL	USA	35	1164	18
Finnerty CC	USA	34	2200	24
Tompkins RG	USA	33	1968	23
Yao YM	China	31	746	16

### Analysis of the top 10 most influential countries/institutions/journals

We ranked the ten highest-output countries/institutions/journals among all authors according to Np (Table 2). The US published the most articles (1193/H:98), followed by China (243/H:31) and Germany (168/H:41). US papers were cited 42,154 times, accounting for 56.45% of the total. This was followed by Germany (5,364) and Canada (4,857). In addition, the US had the highest h-index (98), which was more than twice as high as Germany (41). The relatively low Np but significantly higher h-index and Nc in the UK and Canada compared to China.

**Table 2:** Top ten countries/institutions/journals.

**Subject**		**Np**	**Nc**	**h-index**
Countries				
	USA	1193	42154	98
	China	243	3228	31
	Germany	168	5364	41
	Canada	123	4857	36
	England	112	4822	35
	Japan	106	2803	27
	Australia	79	2530	31
	Turkey	64	892	20
	Italy	62	2458	27
	France	61	4712	29
Organizations				
	University of Texas System (USA)	286	10787	60
	University of Texas Medical Branch Galveston (USA)	188	7368	48
	Shriners Hosp Children (USA)	138	5197	41
	Harvard University (USA)	125	6226	44
	Loyola University Chicago (USA)	110	2899	31
	University of Cincinnati(USA)	88	3294	33
Journals				
	Burns	376	8019	46
	Journal of Burn Care Research	139	1929	23
	Shock	126	4048	35
	Journal of Trauma Injury Infection and Critical Care	83	3098	32
	Critical Care Medicine	75	4757	38
	Journal of Surgical Research	49	988	20
	Annals of Surgery	48	4345	34
	PLOS ONE	43	1203	20
	Archives of Surgery	39	1710	26
	Journal of Burn Care Rehabilitation	36	1255	20

**Figure 3:**
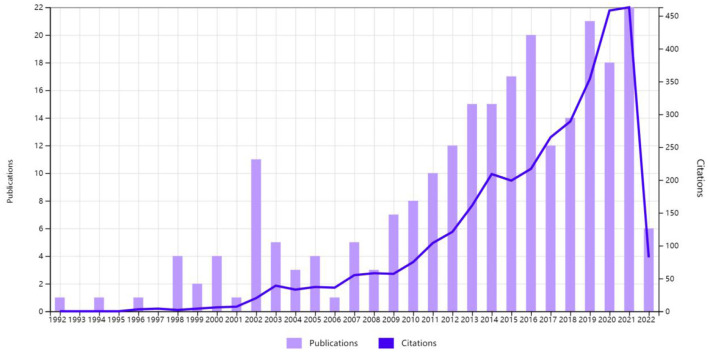
Times cited and publications over time from China.

**Figure 4:**
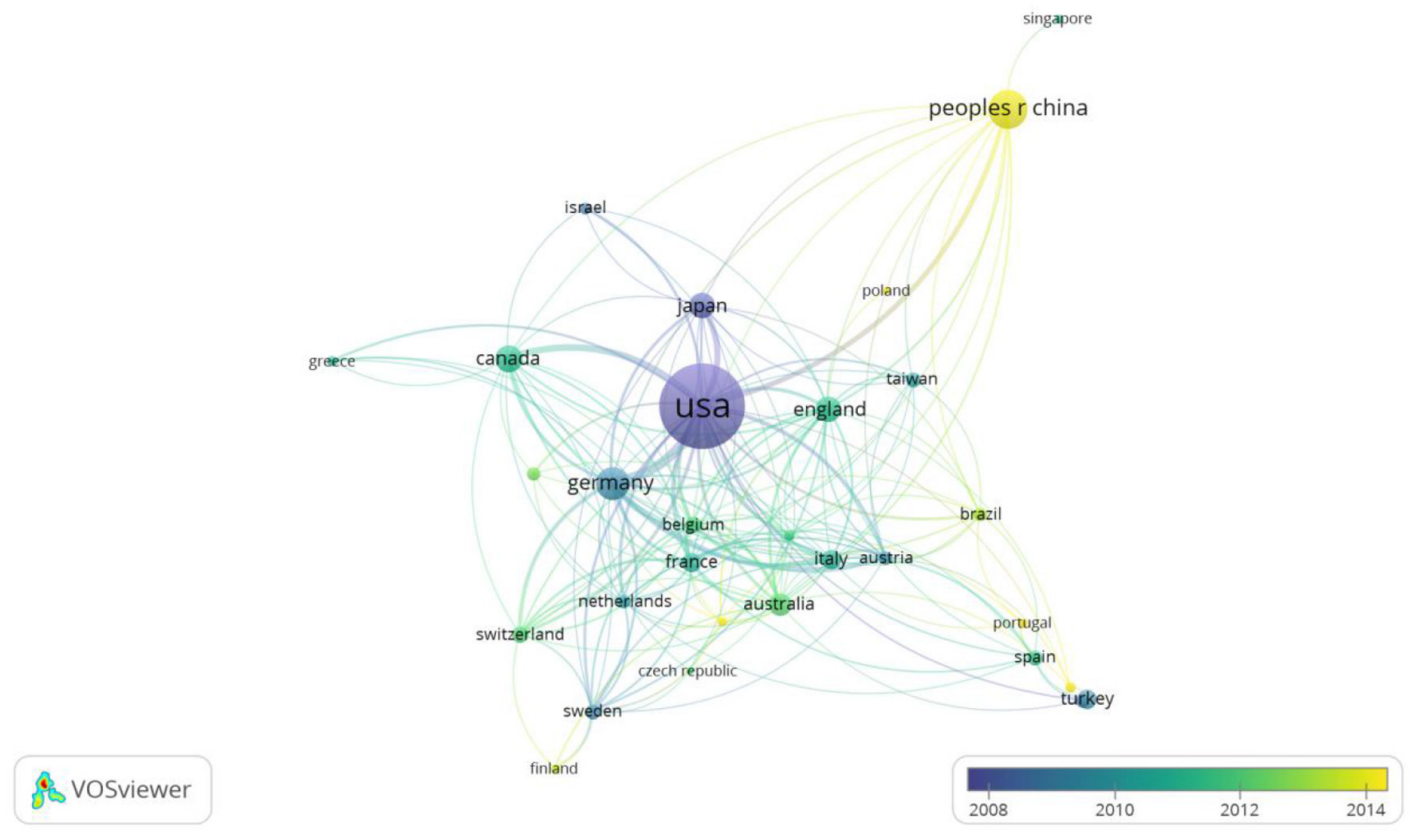
Bibliographic analysis and development of countries with respect to time from more than ten records. Node size represents the number of publications; color represents the average publication year; distance only represents the link strength between two nodes.

Nonetheless, the proportion of Chinese publications and citations in this field has increased rapidly (Figure 3). Meanwhile, we identify from the overlay visualization map (Figure 4) that the US (Total link strength: 224) was the most closely linked country to others in burn sepsis research, reflecting its leadership role in this field. Furthermore, China (Np: 240, APY: 2013. 77) was the most recent major producer of literature in this field, with greater potential for the future.

Table 2 lists the top 10 organizations with the highest number of publications related to burn sepsis. The University of Texas System had the highest Np (286), followed by The University of Texas Medical Branch, Galveston (188), and Shriners Hospitals for Children (138). The University of Texas System was ranked first in Nc (10787) and h-index (60), followed by The University of Texas Medical Branch, Galveston (Nc:7368, H:48). Interestingly, all above organizations belonged to a branch of the University of Texas. The University of Texas was far ahead of the other organizations in all areas. Most of these institutions were located in the US.

Table 2 lists the top 10 journals with the highest number of publications in this field. “Burns” (Np: 376, h-index: 46) published the most papers on burn sepsis, as well as “Journal of Burn Care and Research” (Np: 139, h-index: 23) and “Shock” (Np: 126, h-index: 35), ranked second and third, respectively. Of 2486 documents, about 41% were published in the top 10 academic journals (1014/40.8%). “Critical Care Medicine,” ranked fifth, and “Annals of Surgery,” ranked seventh among the top 10 journals, had higher citation rates and h-indexes.

### Bibliometric analysis of co-citation

**Figure 5:**
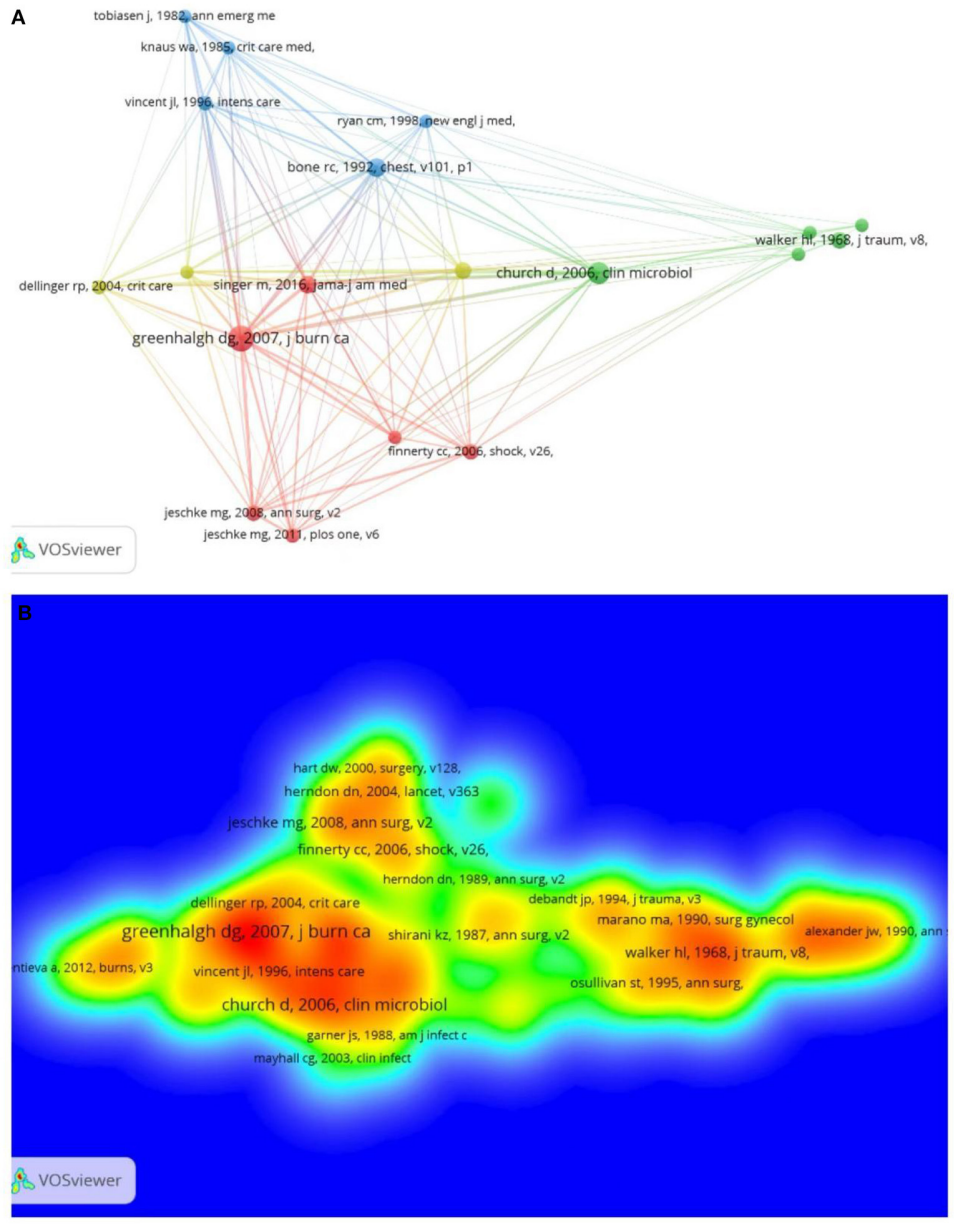
**(A)** Analysis of the co-citation network of cited references: the node's size indicates the frequency of occurrence; the larger the node, the higher the number of references cited. **(B)** Visual analysis of the density of the cited references: the color represents the density of the cited literature. Red indicates a higher frequency of occurrence, while green indicates a lower frequency of occurrence.

The co-citation graph of cited references is displayed in Figure 5A (the references cited 50 times and more were chosen). “The American Burn Association consensus conference defines sepsis and infection in burns” (Greenhalgh dg, 2007) as the most cited literature (192 times). As indicated in the density visualization map (Figure 5B), the highest citation of this article was probably because it provides the diagnostic standard for burn sepsis (ABA criteria) (13). The next most cited paper is “Burn wound infections” (Church d, 2006), with 137 citations.

### Bibliometric analysis of keywords

**Figure 6:**
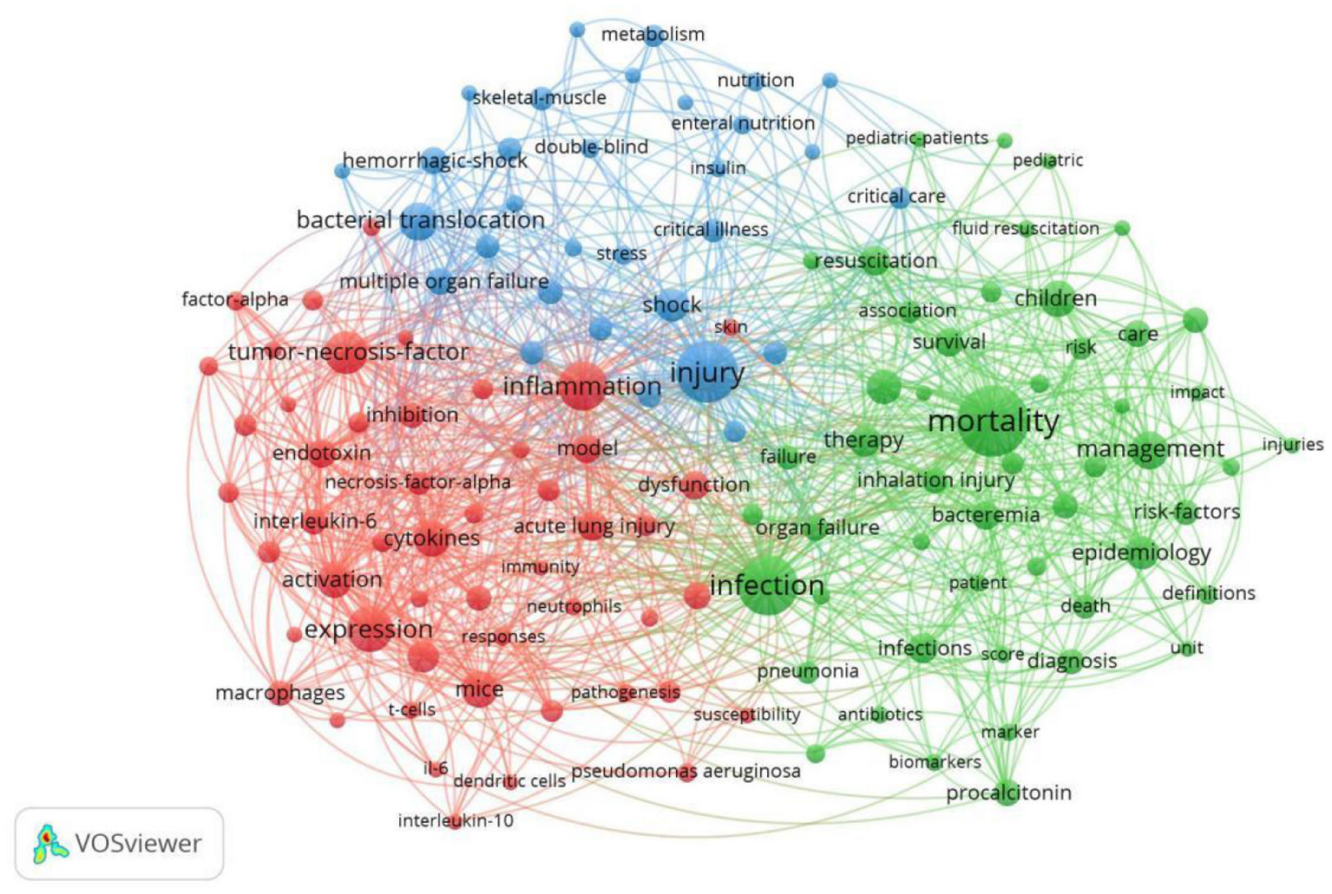
Bibliographic analysis of all keywords in co-occurrence references network map. Based on the relevance of keywords, it can be divided into three different color clusters: cluster one is red, cluster two is green, and cluster three is blue. The size of circles indicates the frequency of occurrence. The larger the nodes, the more frequently the keyword appeared.

Excluding the search terms, synonyms, and duplicate terms (sepsis, burn, burns, septic shock, thermal injury, thermal-injury, severe sepsis, burn injury, and trauma), the keywords extracted from the titles and abstracts of 2,486 papers were analyzed using VOSviewer, and the 133 keywords that appeared more than 25 times were divided into three clusters (Figure 6).

Cluster 1 refers to “studies related to inflammation.” The most frequent keywords were inflammation (178 times), expression (162 times), tumor-necrosis-factor (138 times), cytokines (107 times), and mice (104 times).

Cluster 2 refers to “clinical research.” The most frequent keywords were mortality (349 times), infection (255 times), children (105 times), management (113 times), and critically ill patients (100 times).

Cluster 3 refers to “injury-related studies.” The keywords that appeared more frequently were injury (273 times), bacterial translocation (116 times), shock (90 times), hemorrhagic shock (64 times), and multiple organ failure (64 times).

**Figure 7:**
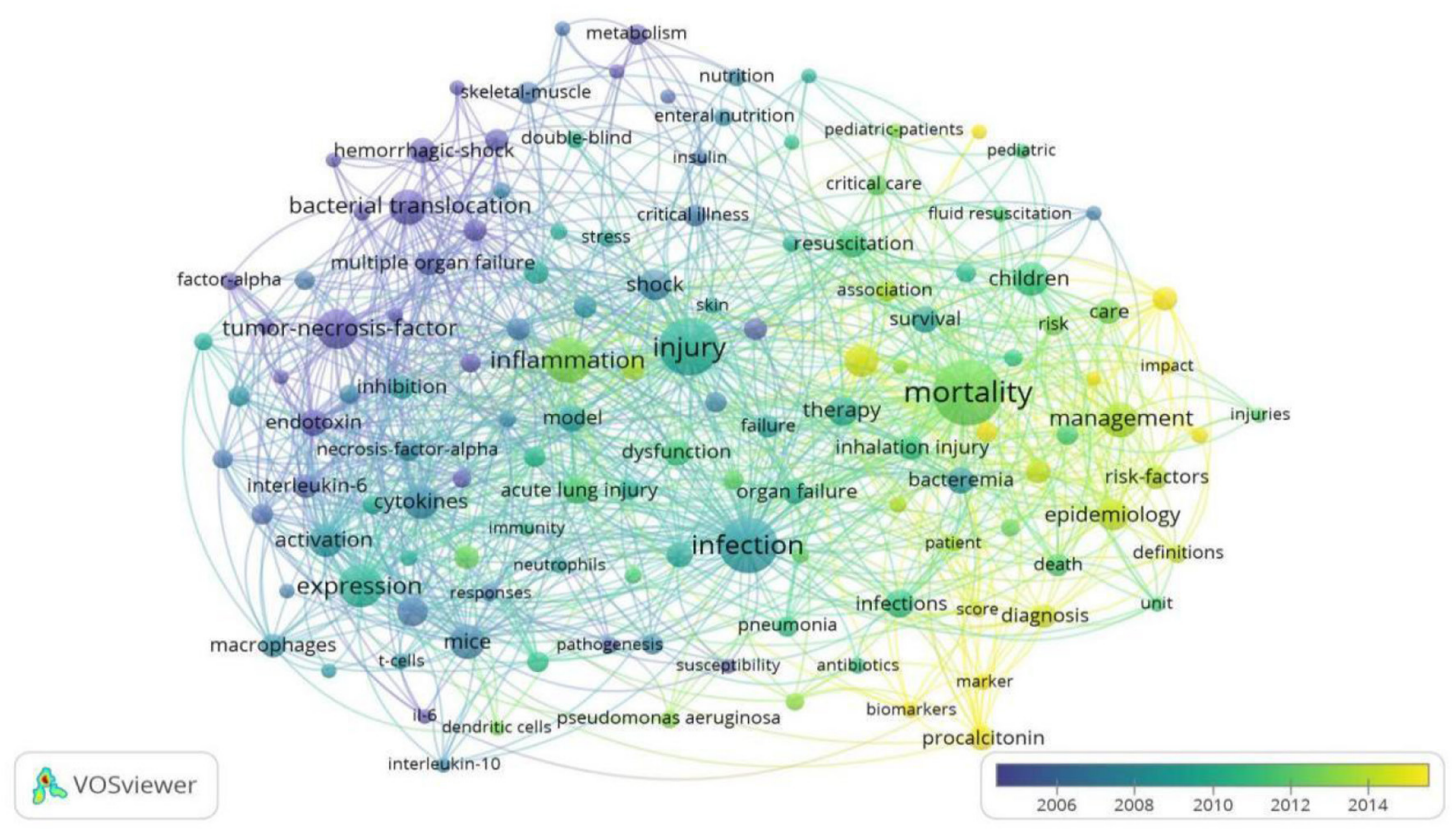
Overlay visualization. Keywords are distributed according to the average time of occurrence. Blue represents the early keywords, and yellow represents the most recent ones. The smaller the distance between two keywords, the more frequently the keywords appear in the same literature simultaneously.

As illustrated in Figure 7, VOSviewer colored all keywords based on the average time of word occurrence. The blue indicates the words that appeared relatively early in the time course, while the yellow indicates recent occurrences. The trend indicates that “clinical research” was the most recent research direction. Among these, “biomarkers” (cluster 2, APY was 2018.16) may be the most recent direction for research in burn sepsis. The most recent keywords in the first cluster (“inflammation-related research”) were “inflammation” (cluster 1, APY was 2012.67), “pseudomonas aeruginosa” (cluster 1, APY was 2012.89), and “dendritic cell” (cluster 1, APY was 2012.42), appearing 178, 37, and 26 times, respectively. As for the third cluster (“injury-related research “): “oxidative stress” (cluster 3, APY was 2013.48) appeared 50 times. In the second cluster (“clinical research”), “acute kidney injury” (cluster 2, APY was 2016.71) appeared 49 times, and it was the most recent keyword besides “biomarkers.”

**Figure 8:**
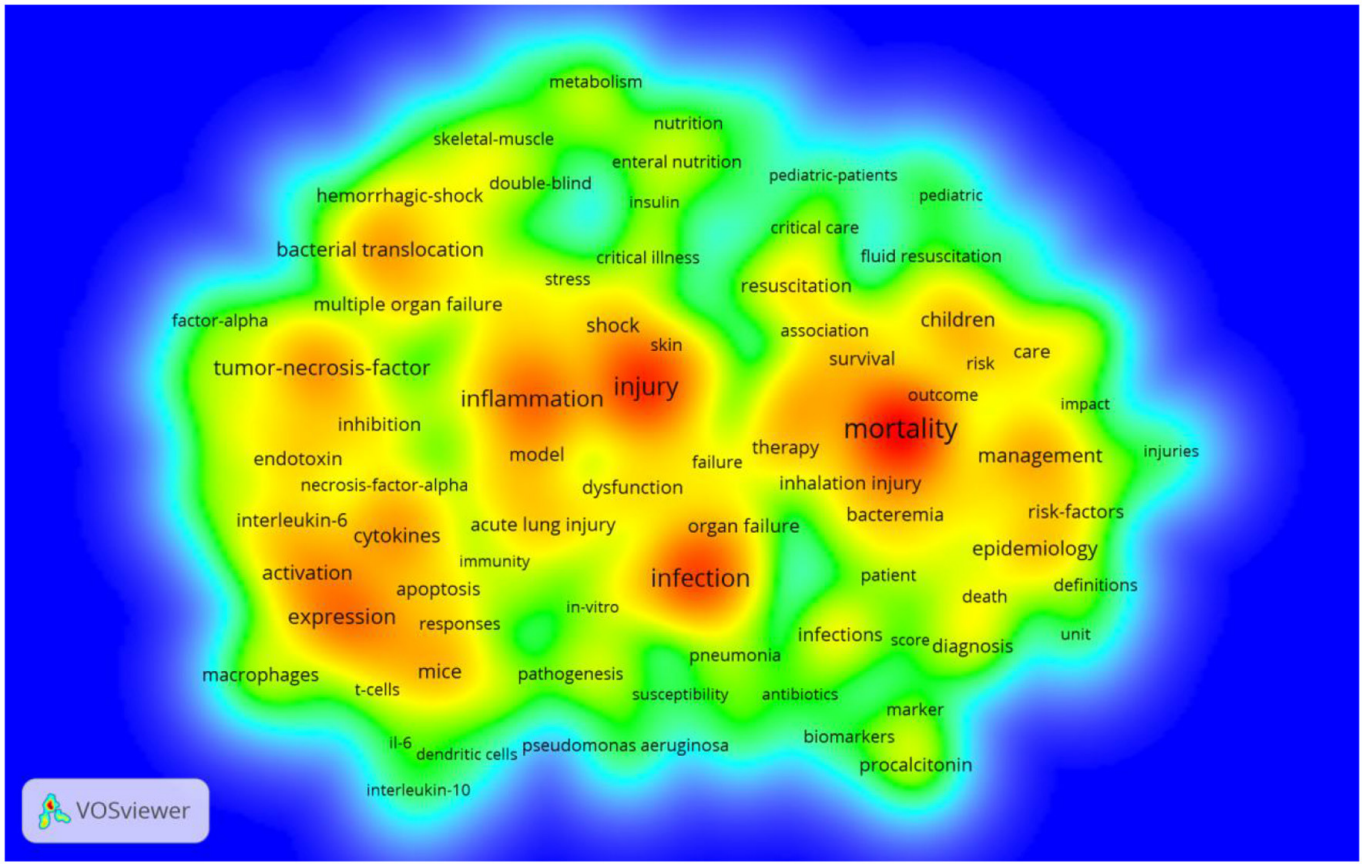
Bibliographic analysis of all keywords in density visualization map. The color represents the density of the keyword. Red represents a more frequent appearance, and green represents a less frequent appearance.

Furthermore, as shown in the visual map of keyword density in Figure 8, the three most hot words were mortality (349 times), injury (273 times), and infection (255 times).

## Discussion

### Trends in the study of burn sepsis

The increase in the overall number of publications indicated that scholars paid increasing attention to burn sepsis research over the last decade. Also, the understanding of burn sepsis was grown. Similarly, the quality of published papers improves, as evidenced by comparing publication growth and citation growth. It is clear from the current study that the US and Germany ranked first and second in the total number of references and h-index values in the burn sepsis research area, respectively. The US has made the most significant contribution to the study of burn sepsis, with the highest number of publications, citation frequency, and h-index.

American clinicians were the first to present the criteria for defining burn sepsis, representing that the US was interested in this research field before the rest of the world. The US has the strongest ties with other countries in this area of research. In addition, conditions for basic medical research and clinical trial study appeared superior in the US. Nine of the top ten influential institutions are located in the US. Moreover, seven of the top ten active authors belong to the US. These characteristics also suggest that the US is leading in this area.

Notably, China ranked second in the total number of published papers but fifth in citation frequency and h-index. The contradiction between the quantity and quality of publications in China may have several causes. The two most important reasons: sepsis diagnosis remains far from standardized in China. In most hospitals in China, even in tertiary hospitals, medical and nursing staff do not regularly perform sepsis-related organ failure assessment (SOFA) scores for critically ill patients, resulting in a high rate of missed sepsis diagnoses. Secondly, China lacks high-quality multicenter randomized clinical trials (RCT) and has a relatively large number of observational studies, which may be insufficient to provide solid evidence in clinical practice. Similarly, Germany, Canada, and the UK have a serious discrepancy between the number of articles and their quality. However, these countries have a high potential for development in this area (Figures 3, 4).

As indicated in Table 2, although Germany, Canada, and England published fewer papers than China from 1981 to 2022, they were more frequently cited and had a higher h-index than China. The US has nine institutions from the top 10 ranking in burn sepsis research, indicating its dominance in this field. The institution with the most publications in this field is the University of Texas. However, it is worth noting that The University of Texas Medical Branch, Galveston, and Shriners Hospitals for Children are all branches of the University of Texas. Although they could also be considered independent organizations because the institutions labeled in the relevant literature are Galveston Hospital and Schreiner Children's Hospital rather than the University of Texas, the key is whether this affiliation affects the results of Np and Nc. We believe it is possible, which may be a shortcoming of the bibliometric analysis. The US has some of the most elite institutions and authors, which partly explains why it has remained a leader in burn sepsis research. In addition, the list includes one Canadian institution. other countries do not have an institution in the top 10 for now. Consequently, more elite other countries' institutions must improve their international position in important research directions related to burn sepsis.

The journal “Burns” has published 376 papers in the field, far ahead of other journals. The remaining journals, including “Journal of Burn Care and Research,” “Shock,” and “Trauma-Injury Infection and Critical Care,” are the leading journals published in the field involving burn sepsis. Therefore, it suggests that future developments in this field may be presented in the journals mentioned above.

As for the authors, Herndon DN from the US and Jeschke MG from Canada published the top two articles on burn sepsis. Herndon DN focuses on clinical research on burn sepsis, including diagnostic criteria and treatment, and his papers have the highest total citation frequency in the list (14). In contrast, Jeschke MG evaluates the potential role of inflammation-related factors in burn sepsis and attempts to understand the pathophysiological response to burn sepsis (15). Herndon DN is a leader in exploring the field of burn sepsis research, and his impressive articles on burn sepsis have been cited extensively. They have made a remarkable contribution to the development of the field (16). In addition, a collaboration between different authors has been significant in studying sepsis and burns. For example, Herndon DN and Jeschke MG, Wolf SE, and Herndon DN have collaborated on research, showing close cooperation between authors and institutions. We believe that these investigators may play a unique and integral role in burn sepsis research, broadly influencing future developments and predicted hot spots in the field.

### Research focuses on burn sepsis

The keyword analysis revealed that most studies in this area were clinical studies, closely followed by basic research on inflammation-related factors. Also, as revealed in the analysis of the cited literature, research has focused on the diagnostic criteria for burn sepsis and its pathophysiological mechanisms. In addition, it began with a consensus conference held by the American College of Chest Physicians and the Society of Critical Care Medicine in 1992 (17) to define sepsis, then progressed to the American Burn Association sepsis criteria (13) and finally to the third international consensus definition of sepsis and septic shock in 2016 (sepsis-3) (18). It is quite clear from these mainstream criteria that the gold standard for diagnosing burn sepsis is yet unclear.

The presence of infection in burn patients is one of the leading causes of sepsis, which in turn is the main cause of death in these patients. In fact, burns are associated with a cascade of events leading to sepsis and multiorgan dysfunction syndromes, such as hypovolemic states, immune and inflammatory reactions, and metabolic changes (19). The incidence of sepsis in burn patients may range from 3 to 30% if the burn area represents more than 20% of the total surface area (TBSA) (20). A more significant concern is that approximately 54% of burn-related deaths result from septic shock and MODS rather than osmotic shock and hypovolemia (21). Recent medical literature has reported that more than 60% of fatalities in burn patients were due to infectious complications and MODS, a direct consequence of sepsis and poor prognosis (22). As a result, early diagnosis and effective treatment of sepsis would benefit burn patients. Besides, mortality was our most frequently used keyword (Figures 7, 8). The most frequent and most popular cited literature is the American burn association consensus conference to define sepsis and infection in burns (Figures 5, 6).

As demonstrated in the mean year of publication analysis of keywords (Figure 8), the most recent research is directed toward clinical studies on “biomarkers” (cluster 2, APY was 2018.16). For promising biomarkers, the potential of some cytokines in the early diagnosis of sepsis after burns has recently been investigated (23). Second, Procalcitonin has been widely studied and clinically applied as a popular biomarker in bacterial infections and sepsis (24). There is growing evidence that presepsin (sCD14-ST) is a promising biomarker for diagnosing sepsis in burn patients. However, it cannot be used alone to confirm or exclude the presence of sepsis in burn patients (25). Mid-regional pro-atrial natriuretic peptide is another promising biomarker (26). In addition, Hampson et al. found that neutrophil function, immature granulocyte counts, and plasma cell-free DNA levels have significant potential in the early diagnosis of sepsis in burn patients (27). It is particularly interesting to observe that miRNA can also be used as a diagnostic biomarker (28). However, no single biomarker can diagnose post-burn sepsis alone, and its values must be interpreted cautiously to ensure an accurate diagnosis.

The heterogeneity of burn patients should be fully considered in the clinical management of sepsis in burn patients (13). Much is known about the pathophysiology of sepsis, which is generally considered an extreme response to inflammation (29). However, burn sepsis has its unique pathogenesis (30), mainly including the following aspects: 1. post-burn infection (trauma infection, inhalation injury, etc.) (31), 2. intestinal flora/endotoxin translocation (32), 3. hypermetabolic state (33), 4. immune dysfunction (34), 5. Other factors include stress response to the neuroendocrine system, coagulation dysfunction, and damage to vital tissues and organs (35). These pathophysiological reactions synergistically induce the development and progression of sepsis and MODS. Accordingly, sepsis treatment in burn patients (36) is broadly divided into the following aspects: 1. fluid resuscitation (37), 2. anti-infection treatment surgical removal of traumatic necrotic tissue, etc. (31, 36), 3. renal replacement therapy (38), 4. immune conditioning strategies (39), 5. adjuvant support and symptomatic treatment, which includes correction of hyperglycemia and electrolyte disorders according to the patient's status; early enteral or parenteral nutrition and reasonable nutritional support; cautious application of glucocorticoids to avoid infection aggravation; and strengthening of adjuvant support therapy for vital organ functions to prevent the occurrence and development of MODS, etc. (40). The diversity of pathogenesis and the lack of recognized diagnostic criteria have prevented the timely and effective treatment of burn sepsis patients. Therefore, based on the massive collection of sepsis patient data, the optimal diagnosis and prognosis prediction model based on different algorithms analyzing patient genetic characteristics, disease history, life history, clinical manifestations, biochemical indicators, treatment response, and so on is the foundation for achieving proper treatment of sepsis in the future (41). The current diagnosis and treatment process for burn sepsis varies between hospitals worldwide, so it is critical to establish a database. Based on establishing a standardized database, bioinformatics professionals with clinical work experience and scientific research ability are required to continuously analyze and revise big data to propose more accurate diagnostic criteria and assessment systems for burn sepsis to truly realize an accurate and intelligent diagnosis and treatment. Therefore, clear diagnostic criteria and predictive biomarkers are essential in preventing and treating burn sepsis. In this regard, establishing a predictive model for early diagnosis, prognosis, and precise treatment of burn sepsis using some reliable indicators (burn area, biomarkers, etc.) may be a hot spot for future research in this field.

### Advantages and limitations

The publications on burn sepsis evaluated in this study were extracted from the Science Citation Index Extended Journals Web of Science database. The data analysis is relatively comprehensive and objective. Nevertheless, some limitations are unavoidable. Due to our inclusion criteria, we only registered publications in English in this survey. Therefore, important studies on burn sepsis research in non-English languages may have been omitted and excluded from the database and analysis. Furthermore, more detailed areas on burn sepsis were not analyzed.

## Conclusion

This study summarized the global research trends regarding burn sepsis over time. The US had made the most significant contribution. Although there are many publications from China, the quality of these papers requires further improvement. The latest research and new developments can be found in Burns and Burn Care Research. Herndon DN, Jeschke MG, Gamelli RL, Horton JW, Wolf SE, and Sheng ZY are good candidates for academic collaboration in this field. Clinically relevant research in burn sepsis has become a hot topic recently, especially in using biomarkers for early diagnosis and prognosis and providing a precise treatment plan.

## Data availability statement

The datasets presented in this study can be found in online repositories. The names of the repository/repositories and accession number(s) can be found in the article/Supplementary material.

## Author contributions

ZC and YZ co-designed this study, conducted the data collection, and wrote the manuscript. J-HL and W-QL participated in the analysis and generated the graphs. XC and J-HZ critically reviewed the original manuscript. All authors have read and approved the manuscript.

## Funding

This study was supported by Science and Technology Program of Jiangxi Province and Department of Health Program of Jiangxi Province, China (grant nos. 20202BBG73020, 20171BBG70061, 202210398, and 2020A0109).

## Conflict of interest

The authors declare that the research was conducted in the absence of any commercial or financial relationships that could be construed as a potential conflict of interest.

## Publisher's note

All claims expressed in this article are solely those of the authors and do not necessarily represent those of their affiliated organizations, or those of the publisher, the editors and the reviewers. Any product that may be evaluated in this article, or claim that may be made by its manufacturer, is not guaranteed or endorsed by the publisher.
